# Has increased telehealth access during COVID-19 led to over-utilization of primary care?

**DOI:** 10.1038/s41746-022-00740-4

**Published:** 2022-12-13

**Authors:** Kaushik P. Venkatesh, Marium M. Raza, Joseph Kvedar

**Affiliations:** Harvard Medical School, Boston, MA USA

**Keywords:** Health care economics, Health policy

## Abstract

Telehealth use for primary care has skyrocketed since the onset of the COVID-19 pandemic. Enthusiasts have praised this new medium of delivery as a way to increase access to care while potentially reducing spending. Over two years into the pandemic, the question of whether telehealth will lead to an increase in primary care utilization and spending has been met with contradictory answers. Some evidence suggests that telehealth may be used as an addition to in-person visits. Others like Dixit et al. have found that telehealth can actually substitute for in-person care rather than contribute to overutilization. As telehealth continues to evolve, outcomes, utilization, and quality of care should be closely monitored.

Telehealth use for primary care has skyrocketed since the onset of the COVID-19 pandemic. Enthusiasts have praised this new medium of delivery as a way to increase access to care while potentially reducing spending. Still, two-plus years into the pandemic, the question of whether telehealth will lead to an increase in primary care utilization and spending has been met with contradictory answers in the scientific literature^1^.

Telehealth involves using a technology-based platform to monitor health or provide medical care. A similar term is telemedicine, which is more clinically focused than telehealth. Telemedicine can involve live virtual discussions between a patient and provider, asynchronous information exchange, virtual consultations between providers in different locations, and remote monitoring of patient health data^2^.

Prior to the COVID-19 pandemic, telehealth adoption was already underway and speculated as a solution to several health care challenges. One key use case is rural populations, where more than a fourth of patients reported difficulty accessing health care in the last year^3^. Telehealth services can provide rural and remote hospitals with timely specialist expertise to increase staff support as well as reduce wait times and delays in care^4^. Some have made the case that telehealth is also more cost-effective. Using telehealth to triage patients appropriately avoids unnecessary emergency department visits, with one study estimating $19–121 net savings per telehealth visit^5^. Finally, telehealth could be particularly effective for older adult patients. It may be easier for patients with mobility restrictions and busy family members to attend follow-up visits virtually, improving health literacy and adherence. Even medication reconciliation can be performed more easily in the virtual context, where medication bottles and pill dispensers already on hand^6^. Studies on telehealth adoption among older adults have focused on the effectiveness of home telehealth programs in chronic disease management and monitoring^7,8^.

With the onset of the COVID-19 pandemic, federal waivers allowed all beneficiaries to receive care through telehealth anywhere it was needed. These waivers removed rural and facility-originating site restrictions and allowed billing to be guided by the level of service, comparable to in-person care^9^. Ultimately, these changes prompted the rapid expansion of telehealth throughout the pandemic, allowing for the provision of care while mitigating infection risk.

Despite all of these potential positive effects, many economists and payers are concerned that increasing access via telehealth will lead to increased utilization of healthcare services^10^.

In this context, Dixit et al. sought to study whether there was a change in primary care utilization with the expanded availability of telehealth^11^. They analyzed 4,114,651 primary care visits from 939,134 unique patients across three healthcare systems between 2019 and 2021. They found that the average number of primary care visits per patient remained stable across patients on commercial insurance, Medicare, and Medicaid. This suggests that the availability of telehealth did not result in additional primary care visits. Instead, telehealth may have served as a substitute for certain in-person encounters. They also found that telehealth use occurred more by patients with multiple primary care visits, suggesting that telehealth was mostly utilized by patients with complex medical needs.

Limitations of the Dixit et al. study include that patients could have received additional care that is not accounted for. Primary care utilization could also have been artificially suppressed due to the pandemic itself, which may not be predictive of telehealth in the post-pandemic era. Separating out the effects of telehealth access and the pandemic independently will be more feasible as new data is collected the as the pandemic subsides. Finally, Dixit et al. did not assess the quality of encounters in telehealth versus in-person settings. In the pursuit of value-based care, it is important to consider the effects of telehealth on the quality of care in primary care, beyond costs and utilization alone.

These findings are in contrast with some recent studies that have shown slightly increased primary care utilization with telehealth availability^12,13^. However, Dixit et al.’s findings are in line with other studies that have found that telehealth reduces primary care spending^1^. These disparities in results may be explained by a variety of reasons. The exact nature of primary care utilization could vary by institution or geographical area—for example, COVID-19 impact and provider response was just one locally variable factor that likely influenced telehealth behavior. Or more simply, some institutes may have used telehealth as a substitute service while others used it as an additive service. For example, some institutional protocols require both in-person and telehealth visits for the management of certain treatments and conditions^14^.

There also remain questions about other unique aspects of telehealth that affect spending. A growing body of evidence has found that telehealth is associated with lower no-show and cancellation rates^15^. No-shows alone cost healthcare providers $150 billion every year—a clear opportunity for telehealth adoption to increase provider revenue and productivity^16^. In addition, telehealth utilization has been associated with lower emergency department utilization and inpatient hospitalizations^15^. In this way, the access-related benefits of telehealth offer new opportunities for primary care to cut down on unnecessary spending and work toward a more streamlined health care system.

Ultimately, policymakers will continue to play a key role in the adoption and utilization of telehealth. The future of parity in telehealth payment rates with payment rates for traditional brick-and-mortar services is still contentious for all players involved—state agencies, CMS, payers, and providers^17^. Such payment considerations will ultimately influence physician behavior. For example, cuts to telehealth rates could put pressure on physicians to switch to in-person service.

Current state waivers that allow for physicians to practice telehealth in another state will also have profound effects on the availability of providers. Currently, 20 states have long-term or permanent interstate telehealth, and 10 have waivers, many of which only have exceptions for COVID-19 patient care^18^. Many other telehealth waivers for provider telehealth privileges and audio-only telehealth are set to expire 151 days after the end of the COVID-19 Public Health Emergency^19^. On July 27, 2022, the House of Representatives passed the Advancing Telehealth Beyond COVID-19 Act of 2021, which extends several COVID-19 telehealth flexibilities through 2024. This bill has not yet cleared the US Senate^20^. The near future of telehealth will be largely shaped by the fate of these waivers over the next few years.

In addition, contrary to common concerns, research suggests that telehealth uptake was actually highest among vulnerable patients^15^. A recent report found the highest rates of telehealth visits were among those with Medicaid (29.3%) and Medicare (27.4%), Black individuals (26.8%), and those earning less than $25,000 (26.7%)^21^. Policy action must continue to leverage primary care telehealth for health equity goals as well; this includes funding for building and modernizing telehealth systems for marginalized communities, regulatory guidelines on accessibility and usability of telehealth platforms, payer coverage of hardware for low-income populations, broadband expansion, public-funded technology support staff, and telehealth private spaces in public facilities like schools and libraries^22^.

Altogether, telehealth is here to stay in primary care and beyond. Some evidence suggests that telehealth may be used as an addition to in-person visits. Others like Dixit et al. have found that telehealth can actually substitute for in-person care rather than contribute to overutilization. This finding is an argument to continue expanding telehealth for its access benefits, particularly for vulnerable populations. Further research is needed to understand the factors affecting telehealth adoption by primary care providers. As telehealth continues to evolve, outcomes, utilization, and quality of care should be closely monitored. Such efforts will provide regulators and payers with the data to incentivize appropriate utilization behaviors. Finally, telehealth is inherently political in the current healthcare ecosystem. Federal and state policies like payment parity and provider licensure will largely shape the future of telehealth.

## Data Availability

No datasets were produced or analyzed for this article.

## Code availability

No computer code was produced or analyzed for this article.
